# Genotyping, antibiotic resistance and prevalence of *Arcobacter* species in milk and dairy products

**DOI:** 10.1002/vms3.800

**Published:** 2022-04-14

**Authors:** Abazar Lameei, Ebrahim Rahimi, Amir Shakerian, Hassan Momtaz

**Affiliations:** ^1^ Department of Food Hygiene Shahrekord Branch Islamic Azad University Shahrekord Iran

**Keywords:** Arcobacter species, dairy products, milk

## Abstract

**Background:**

*Arcobacter* spp. has been considered an emerging foodborne pathogen and a hazard to human health. The dairy chain has been isolated from different sources; nevertheless, data on *Arcobacter* occurrence in raw milk and dairy products in Iran are still scant.

**Objective:**

The present study investigates the prevalence, antimicrobial susceptibility and the presence of virulence genes of *Arcobacters* species isolated from milk and dairy products.

**Methods:**

Then, a total of 350 raw milk samples and 400 dairy product samples were collected from dairy supply centers in Isfahan, Iran. Presumptive *Arcobacter* strains were obtained by enriching samples in Oxoid *Arcobacter* enrichment broth (AEB) followed by the filtration of enrichment product through 0.45‐μm pore size membrane filters laid onto non‐selective blood at 30°C under microaerophilic conditions. Molecular identification of *Arcobacter cryaerophilus* and *A. butzleri* was performed by Polymerase chain reaction (PCR) amplification of the 16S rRNA gene, followed by sequencing. The disc diffusion method was used to determine the antimicrobial susceptibility of isolates. Targeted resistance and virulence genes were detected using multiplex PCR.

**Results:**

The results show a low recovery rate of *Arcobacter* spp. in milk. *Arcobacters* were found in all types of milk, except raw camel milk, but were absent from all dairy products. *Arcobacter butzleri* was the predominant species in raw milk. Detection of virulence genes shows that all virulence genes targeted were found among *A. butzleri*, and six (*cadF*, *cj1349*, *irgA*, *mviN*, *pldA*, *tlyA*) were found among *A. cryaerophilus*. All *A. butzleri* strains and some *A. cryaerophilus* strains isolated from milk were resistant to amoxicillin‐clavulanic acid and tetracycline. All *A. cryaerophilus* isolates from milk were susceptible to gentamycin, streptomycin, erythromycin and ciprofloxacin. The distribution of resistance genes in *Arcobacter* strains in milk shows that all isolates carried *tet(O)* and *bla_OXA‐61_* genes.

**Conclusions:**

In conclusion, the results indicate a low recovery rate of *Arcobacter* spp. in milk and milk products. However, a significant number of *Arcobacter* strains with putative virulence genes may be potential pathogens for humans and an overall increase in *Arcobacter* resistance to first‐line antibiotics. These results highlight the need for regular surveillance of *Arcobacter* strains in milk and milk products in Iran.

## Graphical abstract

The results indicate a low recovery rate of *Arcobacter* spp. in milk and milk products. However, there was a significant number of *Arcobacter* strains with putative virulence genes that may be potential pathogens for humans and an overall increase in *Arcobacter* resistance to first‐line antibiotics. These results highlight the need for regular surveillance of the occurrence of *Arcobacter* strains in milk and milk products in Iran.

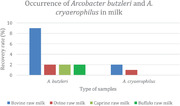

## 1 INTRODUCTION

*Arcobacter* is curved to S‐shaped rod gram‐negative bacilli, motile, non‐spore‐forming, typically 0.2–0.9‐mm wide and 0.5—3‐mm long. They are facultative aerobic‐anaerobic and can survive between 15 and 42°C. Microaerobic conditions (3%–10% O_2_) are recommended for optimal growth (Ho et al., 2006). The genus *Arcobacter* belongs to the *Campylobacteraceae* family and consists of six species: *Arcobacter nitrofigilis, A. butzleri, A. cryaerophilus, A. skirrowii, A. cibarius* and *A. halophilus* (Donachie et al., 2005; Houf et al., 2005; Vandamme & De Ley, 1991).

*Arcobacter* species are considered important food and water‐borne pathogens (Shah et al., 2011). *Arcobacter* spp. commonly enter food production through faecal contamination from various sources (Öngör et al., 2004; Scullion et al., 2006; Serraino et al., 2013). Several studies have reported the presence of *Arcobacter* species in various types of food samples, including vegetables (González & Ferrús, 2011; González et al., 2017; Mottola et al., 2016), meats (Lehmann et al., 2015; Rahimi, 2014; Rivas et al., 2004), shellfish (Leoni et al., 2017; Levican et al., 2014; Mottola et al., 2016), fish (Laishram et al., 2016), eggs (Lee et al., 2016) and drinking water (Ertas et al., 2010; Jacob et al., 1998; Jalava et al., 2014). *Arcobacter* species can be pathogens, opportunistic pathogens and commensals associated with human and animal diseases (Ho et al., 2006). The consumption and handling of raw or poorly cooked foods of animal origin are the main routes of transmission of *Arcobacters* to humans (Giacometti et al., 2014; Shah et al., 2012; Van Driessche et al., 2005). *Arcobacter butzleri, A. cryaerophilus* and *A. skirrowii* are responsible for gastrointestinal diseases with persistent diarrhoea, enterocolitis, peritonitis and bacteremia in humans (Collado & Figueras, 2011; Jiang et al., 2010; Lappi et al., 2013; Mottola et al., 2016; Webb et al., 2016), while in animals, they can trigger gastroenteritis, mastitis, bacteremia and reproductive disorders (Arguello et al., 2015; Ho et al., 2006; Logan et al., 1982; Van Driessche & Houf, 2008).

Regarding dairy animals, *Arcobacters* have been widely reported to be isolated (Piva et al., 2013; Shah et al., 2013) and have been found in various sources, including raw milk and fresh cheese (Ertas et al., 2010; Shah et al., 2012; Yesilmen et al., 2014). Due to the complexity of operations in the dairy production chain, *Arcobacter* contamination can occur in several ways (Giacometti et al., 2014). Indeed, *Arcobacters* have been found in bulk milk tanks (Elmali & Can, 2017; Ertas et al., 2010), milking equipment, barn floors, inline filters in milking machinery and cheese (Giacometti et al., 2013, 2015; Serraino et al., 2013). In Iran, the dairy sector is one of the leading traditional sectors, and economic activities and milk production have increased to a level of about 9 billion kg of milk per year (Beldman et al., 2017). With the high demand, the sale of raw milk for direct consumption may have increased human exposure to zoonotic agents (Haran et al., 2012). Numerous studies in Iran recovered *Arcobacters* species from animal products (Khodamoradi & Abiri, 2020; Rahimi, 2014; Shirzad Aski et al., 2016), but data about the occurrence of *Arcobacters* in milk and dairy products in Iran are scant. In addition, the isolation of resistant *Arcobacter* species from animal products with virulent and pathogenic determinants has been increasingly reported (Goojani et al., 2020.; Karadas et al., 2013; Sekhar et al., 2017; Tabata, 2014). In this respect, the present study investigates the prevalence, antimicrobial susceptibility and presence of virulence genes of *Arcobacter* species isolated from milk and dairy products collected from dairy supply centres in Isfahan, Iran.

## 2 MATERIALS AND METHODS

### 2.1 Sampling

Samples analysed in the current study were collected randomly from dairy supply centres in Isfahan, Iran. The samples consisted of raw milk from various animals (bovine, ovine, caprine, buffalo and camel) and traditional dairy products (cheese, cream, butter and ice cream). All samples were aseptically collected in separate sterile plastic bags to avoid cross‐contamination and were kept in a cooler with ice packs until they arrived at the laboratory for microbiological analysis. A total of 350 raw milk samples and 400 dairy product samples were collected.

### 2.2 Isolation of *Arcobacters*

Isolation of *Arcobacters* was performed following the method described by Atabay et al. (2003). Samples were mixed using a vortex mixer at room temperature. Then, 10 ml was homogenised with 90 ml of AEB (Oxoid) plus cefoperazone, amphotericin B and teicoplanin (Oxoid selective supplement) and incubated at 30°C for 48 h under microaerophilic conditions. After incubation, 300 μl of each enriched sample was transferred to a cellulose acetate membrane filter (Filterlab) with a pore size of 0.45 μM. After 1 h of passive filtration (30°C, aerobic conditions), the filters were aseptically removed, and the plates were incubated at 30°C under microaerobic conditions. Plates were checked every 24 h (up to 7 days) for the presence of typical *Arcobacter* colonies. From each plate, five suspect colonies were subcultured onto Mueller Hinton Broth (MHB) plates for 48 h at 30°C under microaerobic conditions.

### 2.3 Molecular identification of *Arcobacters*

Template DNA was extracted from presumptive *Arcobacter* isolates using PrepMan Ultra Reagent (Applied Biosystems) following the manufacturer's instructions. Molecular identification of *A. cryaerophilus* and *A. butzleri* was performed by amplification of the 16S rRNA gene using PCR, followed by sequencing. The resulting sequence was compared to known sequences of the 16S rRNA gene in GenBank by multiple sequence alignment (Lau et al., 2002).

### 2.4 Antibiotic susceptibility testing

The disc diffusion method on Mueller–Hinton agar was used to test the antibiotic susceptibility of isolates to tetracycline (30 μg/disk), streptomycin (10 μg/disk), gentamycin (10 μg/disk), cephalothin (30 μg/disk), ciprofloxacin (5 μg/disk), ampicillin (10u/disk), amoxicillin‐clavulanic acid (20/10u/disk), cefotaxime (30 μg/disk), nalidixic acid (30 μg/disk) and erythromycin (15 μg/disk).

### 2.5 Detection of virulence and resistance genes

A total of nine virulence genes (*cadF, ciaB, cj1349, irgA, hecA, hecB, mviN, pldA, tlyA*) and four resistance genes (*tet(O), cmeB, bla_OXA‐61,_ aphA‐3‐1*) were identified. The PCR mixture contained 2‐μl template DNA, 12.5 μl of DreamTaq Green PCR Master Mix (2x) (Thermo Fisher Scientific), 1 μM of each primer, 0.5 μM of primer SKIR F and 8.25 μl of molecular grade water (Thermo Fisher Scientific) in a total reaction volume of 25 μl. The PCR conditions consist of an initial denaturation step at 94°C for 2 min. This step was followed by 32 PCR cycles, consisting of denaturation at 94°C for 45 s, annealing (variable) for 45 s, extension at 72°C for 30 s and a final elongation step at 72°C for 5 min (Douidah et al., 2012). DNA fragments were analysed by electrophoresis in a 2% agarose gel stained with ethidium bromide. The 100 bp DNA ladder was used as the molecular weight marker. Interpretation of the results was made based on comparing the migration of the fragments to the marker sizes. The list of genes detected in this study is presented in Table 1.

**TABLE 1:** Sequences and positions of the primers designed for the detection of *the Arcobacter* and virulence and resistance genes

**Target gene**	**Sequence of primers (5′‐3′)**	**Amplicon size (bp)**	**Reference**
*16SrRNA*	F: AGTTTGATCCTGGCTCAG R: AGGCCCGGGAACGTATTCAC	1414	(Lau et al., 2002)
*cadF*	F: TTACTCCTACACCGTAGT R: AAACTATGCTAACGCTGGTT	283	(Douidah et al., 2012)
*ciaB*	F: TGGGCAGATGTGGATAGAGCTTGGA R: TAGTGCTGGTCGTCCCACATAAAG	284	(Douidah et al., 2012)
*cj1349*	F: CCAGAAATCACTGGCTTTTGAG R: GGGCATAAGTTAGATGAGGTTCC	659	(Douidah et al., 2012)
*irgA*	F: TGCAGAGGATACTTGGAGCGTAACT R: GTATAACCCCATTGATGAGGAGCA	437	(Douidah et al., 2012)
*hecA*	F: GTGGAAGTACAACGATAGCAGGCTC R: GTCTGTTTTAGTTGCTCTGCACTC	537	(Douidah et al., 2012)
*hecB*	F: CTAAACTCTACAAATCGTGC R: CTTTTGAGTGTTGACCTC	528	(Douidah et al., 2012)
*mviN*	F: TGCACTTGTTGCAAAACGGTG R: TGCTGATGGAGCTTTTACGCAAGC	294	(Douidah et al., 2012)
*pldA*	F: TTGACGAGACAATAAGTGCAGC R: CGTCTTTATCTTTGCTTTCAGGGA	293	(Douidah et al., 2012)
*tlyA*	F: CAAAGTCGAAACAAAGCGACTG R: TCCACCAGTGCTACTTCCTATA	230	(Douidah et al., 2012)
*tet(O)*	F: GCGTTTTGTTTATGTGCG R: ATGGACAACCCGACAGAAG	559	(Gharbi et al., 2021)
*cmeB*	F: TCCTAGCAGCACAATATG R: AGCTTCGATAGCTGCATC	241	(Forson et al., 2020)
*bla_OXA‐61_*	F: AGAGTATAATACAAGCG R: TAGTGAGTTGTCAAGCC	372	(Forson et al., 2020)
*aphA‐3‐1*	F: TGCGTAAAAGATACGGAAG R: CAATCAGGCTTGATCCCC	701	(Forson et al., 2020)

## 3 RESULTS

Table 2 shows the results for the presence of *Arcobacter* spp. in milk and milk products. In general, few samples produced *Arcobacter* colonies. *Arcobacters* were present in all types of milk, except in raw camel milk, but absent in all dairy products.

**TABLE 2:** Occurrence of *Arcobacter* spp. in milk and dairy products

**Type of samples**	**Number of samples**	**Occurrence of *Arcobacter* spp. (%)**
Bovine raw milk	120	11
Ovine raw milk	60	3
Caprine raw milk	100	2
Buffalo raw milk	32	2
Camel raw milk	38	–
**Total milk**	**350**	18
Traditional cheese	100	–
Traditional cream	100	–
Traditional butter	100	–
Traditional ice cream	100	–
**Total dairy products**	**400**	–
**Total**	**750**	18

**FIGURE 1:**
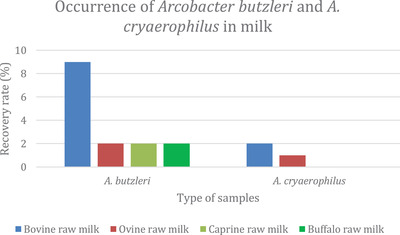
Occurrence of *Arcobacter butzleri* and *A. cryaerophilus* in milk

Figure 1 presents the occurrence of *A. butzleri* and *A. cryaerophilus* in milk. The results show that *A. butzleri* was found in each type of milk sample, while *A. cryaerophilus* was present in raw bovine and ovine milk.

**FIGURE 2:**
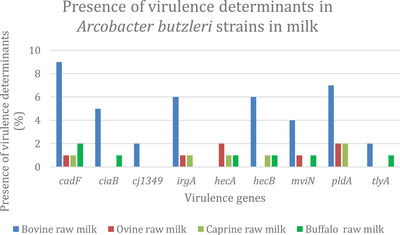
Presence of virulence determinants in *A. butzleri* strains in milk

Figure 2 presents the results regarding virulence determinants in *A. butzleri* strains in milk. The results show that *A. butzleri* isolated from all samples carried the *cadF* gene. *A. butzleri* isolated from bovine raw milk carried all virulence genes targeted except *hecA*. Concerning ovine raw milk, both isolates had *hecA* and *pldA* genes. *CiaB, cj1349* and *tlyA* were absent in *A. butzleri* isolates from ovine and caprine raw milk. *Cj1349, irgA* and *pldA* were not detected in *A. butzleri* isolates from raw buffalo milk.

**FIGURE 3:**
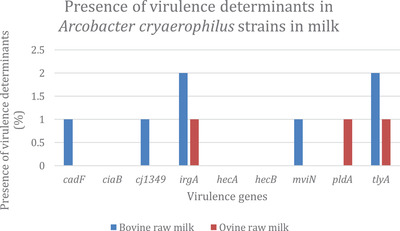
Presence of virulence determinants in *A. cryaerophilus* strains in milk

Figure 3 shows the presence of virulence determinants in *A. cryaerophilus* strains in milk. The results show that all isolates carried the *irgA* and *tlyA* genes. The *CiaB, hecA* and *hecB* genes were absent in all *A. cryaerophilus* isolates. In addition, none of *A. cryaerophilus* isolated from bovine raw milk carried the *pldA* gene, and none from ovine had the *cadF cj1349* and *mviN* genes.

Table 3 shows an antimicrobial pattern of *A. butzleri* strains isolated from milk. All *A. butzleri* strains isolated from milk were resistant to amoxicillin‐clavulanic acid and tetracycline. At least one isolate from bovine raw milk exhibited resistance to each antibiotic tested. All *A. butzleri* isolates from ovine and caprine raw milk were resistant to cephalothin.

**TABLE 3:** Antimicrobial resistance properties in *Arcobacter butzleri* strains in milk

***A. butzleri* (%)**	**GM10**	**S10**	**AM10**	**AMC20/10**	**CF30**	**CTX30**	**NA30**	**TE30**	**E15**	**CIP5**
Bovine raw milk (9)	1	2	9	8	7	7	6	9	1	1
Ovine raw milk (2)	–	1	2	1	2	–	2	2	1	1
Caprine raw milk (2)	–	–	2	1	2	–	1	2	–	1
Buffalo raw milk (2)	–	–	2	–	1	–	–	2	–	–
**Total (15)**	**1**	**3**	**15**	**10**	**12**	**7**	**9**	**15**	**2**	**3**

*Note*: S10, (10 μg/disk); AM 10, ampicillin (10 u/disk); AMC 20/10, amoxicillin‐clavulanic acid (20/10 u/disk); CF 30, cephalothin (30 μg/disk); CIP 5, ciprofloxacin (5 μg/disk); CTX 30, cefotaxime (30 μg/disk); E 15, erythromycin (15 μg/disk); GM 10, gentamycin (10 μg/disk); NA 30, nalidixic acid (30 μg/disk); TE 30, tetracycline (30 μg/disk).

**FIGURE 4:**
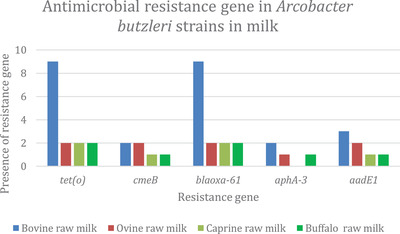
Antimicrobial resistance genes in *A. butzleri* strains in milk

Figure 4 shows the distribution of resistance genes in *A. butzleri* strains in milk. The results show that all isolates carried the *tet(O)* and *bla_OXA‐61_* genes. All targeted resistance genes were found in isolates from bovine raw milk. The *cmeB* gene was present in both isolates from ovine raw milk, and no isolates from caprine raw milk carried *aphA‐3*.

Table 4 shows an antimicrobial pattern of *A. cryaerophilus* strains in milk. All *A. cryaerophilus* isolates from milk were susceptible to gentamycin, streptomycin, erythromycin and ciprofloxacin. All isolates from bovine raw milk were resistant to amoxicillin‐clavulanic acid, cephalothin, cefotaxime and tetracycline. The isolate from ovine raw milk was resistant to ampicillin, cephalothin, nalidixic acid and tetracycline.

**TABLE 4:** Antimicrobial resistance properties in *A. cryaerophilus* strains in milk

***A. cryaerophilus* (%)**	**GM10**	**S10**	**AM10**	**AMC20/10**	**CF30**	**CTX30**	**NA30**	**TE30**	**E15**	**CIP5**
Bovine raw milk (2)	–	–	1	2	2	2	1	2	–	–
Ovine raw milk (1)	–	–	1	–	1	–	1	1	–	–
**Total (3)**	**–**	**–**	**2**	**2**	**3**	**2**	**2**	**3**	**–**	**–**

*Note*: S10, (10 μg/disk); AM 10, ampicillin (10 u/disk); AMC 20/10, amoxicillin‐clavulanic acid (20/10 u/disk); CF 30, cephalothin (30 μg/disk); CIP 5, ciprofloxacin (5 μg/disk); CTX 30, cefotaxime (30 μg/disk); E 15, erythromycin (15 μg/disk); GM 10, gentamycin (10 μg/disk); NA 30, nalidixic acid (30 μg/disk); TE 30, tetracycline (30 μg/disk).

**FIGURE 5:**
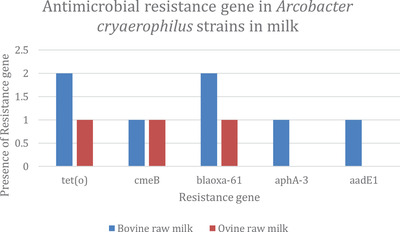
Antimicrobial resistance genes in *A. cryaerophilus* strains in milk

Figure 5 shows the distribution of resistance genes in *A. cryaerophilus* strains in milk. All *A. cryaerophilus* isolates from milk carried the *tet(O)* and *bla_OXA‐61_* genes. All targeted resistance genes were found in isolates from bovine raw milk. None of the isolates from ovine raw milk had *aphA‐*3 and *aadE1*.

**FIGURE 6:**
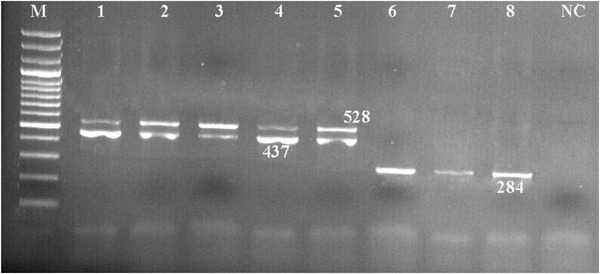
Results of the PCR assay for the identification of virulence genes in *Arcobacter* isolates. M: DNA size ladder 100 bp (Fermentas), lane NC: negative control; lane 1–8: positive samples (*irgA, hecB, ciaB* genes)

**FIGURE 7:**
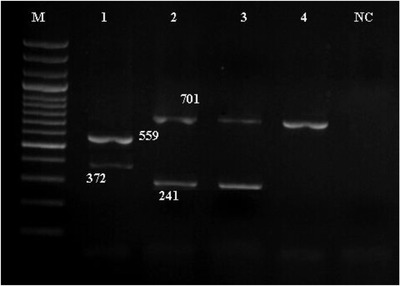
Results of the PCR assay for the identification of resistance genes in *Arcobacter* isolates. M: DNA size ladder 100 bp (Fermentas), lane NC: negative control; lane 1–4: positive samples (*blaOXA‐61, tet(O), aphA‐3‐1, cmeB* genes)

Figures 6 and 7 show the results of the PCR assay for the identification of 16S rRNA genes, virulence genes and resistance genes in *Arcobacter* isolates.

## 4 DISCUSSION

*Arcobacter* spp. is related to human and animal disease, and it is considered an emerging serious foodborne pathogen (Collado & Figueras, 2011). The present study aims to assess the prevalence and characteristics of *Arcobacter* spp. isolated from milk and dairy products collected from dairy supply centers in Isfahan, Iran.

The presence of *Arcobacter* spp. in milk and dairy products shows that few samples produced *Arcobacter* colonies. *Arcobacters* were present in all types of milk, except raw camel milk, but were absent from all dairy products. Several studies have reported the presence of *Arcobacters* in milk but also in dairy products, including cheese (Giacometti et al., 2013; Serraino & Giacometti, 2014; Yesilmen et al., 2014). Numerous factors, including the experimental design, sample size and identification/isolation method used, influence the recovery rate in field studies of *Arcobacter* spp. or specific *Arcobacter* species in animals and animal products (Ho et al., 2006). Pasteurisation or sterilisation of milk before processing into dairy products may explain the absence of *Arcobacters* in the collected dairy products. The absence of *Arcobacters* in raw camel milk is consistent with Goojani et al. (2020), who did not isolate any *Arcobacter* spp. from camel meat collected in Iran. Camel milk is one of the primary sources of animal milk in Africa and the Arab region (Watson & Preedy, 2019). Still, to date, no study has reported the presence of *Arcobacters* in this milk. *Arcobacter butzleri* was the most isolated species in raw milk and was found in each type of milk sample, while *A. cryaerophilus* was present in bovine and ovine raw milk. This finding is in line with some research showing that *A. butzleri*, followed by *A. cryaerophilus*, are the most commonly found species in milk and dairy products (Giacometti et al., 2013; Serraino & Giacometti, 2014; Yesilmen et al., 2014). *Arcobacter butzleri* is a pathogen responsible for diarrhoea and septicemia in humans and is frequently isolated from milk and dairy products (Parisi et al., 2019). It is the most recovered species because it has an inherent ability to survive in different environments and under extremely harsh conditions (Badilla‐Ramírez et al., 2016; Giacometti et al., 2015; Ramees et al., 2017). In addition, *Arcobacter* species, including *A. cryaerophilus* and *A. skirrowii*, are more susceptible to antimicrobials and other components used in isolation media, making them more difficult to isolate (Atabay et al., 1998; Houf et al., 2001).

Knowledge about virulence factors affecting the pathogenicity of *Arcobacter* species is still limited. Characterisation of virulence determinants would help to establish a pathogenic profile of the isolated *Arcobacter* species (Goojani et al., 2020). Detection of virulence genes showed that all virulence genes targeted were found among *A. butzleri*, and six genes (*cadF, cj1349, irgA, mviN, pldA, tlyA*) were found among *A. cryaerophilus*. All *A. butzleri* isolates carried the *cadF* gene, while all *A. cryaerophilus* isolates carried the *irgA* and *tlyA* genes. The genes detected in *A. butzleri* (*cadF*, *pldA, irgA, hecB, ciaB, mviN*) have also been detected in numerous studies (Ferreira et al., 2014; Girbau et al., 2015; Karadas et al., 2013; Laishram et al., 2016; Mottola et al., 2016; Piva et al., 2017; Tabata, 2014; Zacharow et al., 2015). Girbau et al. (2015), Tabata (2014) and Zacharow et al. (2015) also found virulence genes in *A. cryaerophilus* isolates (*irgA*, *tlyA, pldA, mviN, cj1349, cadF)* as in this study. The difference concerning the most frequently detected genes in our study and the other studies can be explained by the small number of isolates obtained and studied in our research. Ten putative virulence genes (*cadF*, *mviN*, *pldA*, *tlyA*, *cj1349*, *hecB*, *irgA*, *hecA*, *ciaB* and *iroE;* Miller et al., 2007) have been identified in *Arcobacters*, but it is not yet known whether these genes encode similar functions to their homologs in other species (Lehmann et al., 2015). The *ciaB, mviN, tlyA, cj1349, pldA *and* cadF* genes code for adhesion and invasion mechanisms, and *hecA* and *hecB* code for hemolysin secretion (Piva et al., 2017). However, the contribution of these genes in each strain needs to be elucidated through both in vitro and in vivo approaches (Kim et al., 2019).

Determination of antimicrobial resistance patterns is vital for a better choice of antibiotic as a first‐line drug for treating *Arcobacter* infection (Houf et al., 2004; Vandenberg et al., 2006). In the present study, all *A butzleri* strains and some *A. cryaerophilus* strains isolated from milk were resistant to amoxicillin‐clavulanic acid and tetracycline. This is not the case in the study by Elmali and Can (2017), who found tetracycline to be the most effective antibiotic. Similar to our study, several authors found some isolates exhibiting resistance to gentamycin (Elmali & Can, 2017), streptomycin and tetracycline (Goojani et al., 2020), cephalothin (Atabay & Aydin, 2001; Rahimi, 2014), erythromycin and ciprofloxacin (Atabay & Aydin, 2001; Son et al., 2007) and ampicillin (Shah et al., 2013). All *A. cryaerophilus* isolates from milk were susceptible to gentamycin, streptomycin, erythromycin and ciprofloxacin. This result follows those obtained by Vidal‐Veuthey et al. (2021), who reported that all *Arcobacter* strains were susceptible to four antibiotics evaluated in his study, including erythromycin and ciprofloxacin, tetracycline and gentamicin. Differences in the susceptibility patterns could be explained by the frequency of drugs in animals for treatment and/or prophylaxis, the lack of standardisation for *Arcobacter* antimicrobial susceptibility tests and the absence of established breakpoints (Rahimi, 2014). The distribution of resistance genes in *Arcobacter* strains in milk show that all isolates carried *tet(O)* and *bla_OXA‐61_* genes. This indicates that tetracycline and beta‐lactams are frequently used antibiotics in dairy animal production. The presence of the *tet(o)* gene in all *Arcobacter* strains isolated from milk is consistent with the resistance of these strains to the antibiotic tetracycline (Connell et al., 2003). The high resistance observed among *Arcobacter* strains to beta‐lactam antibiotics, including amoxicillin and ampicillin, is confirmed by the presence of the *blaOXA‐61* genes encoding beta‐lactamase production in all isolates (Forson et al., 2020). The *CmeB* gene present in some strains may confer the resistance observed in some isolates to several antibiotics by decreasing porin expression (Cagliero et al., 2006). The presence of the *aadE* gene in *Arcobacters* highlights the possibility of genetic transfer of information from gram‐positive to gram‐negative bacteria, which explains the rarely observed resistance to antibiotics such as gentamycin (Pinto‐Alphandary et al., 1990).

All targeted resistance genes in some isolates are due to the accumulation of many antibiotic resistance genes by *Arcobacters* species (Millar & Raghavan, 2017).

## 5 CONCLUSION

*Arcobacter* species are emerging human pathogens of animal origin. The current study shows a low recovery rate of *Arcobacter* spp. in milk and their absence in dairy products. Pasteurisation or sterilisation of milk before processing into dairy products effectively reduces the occurrence of *Arcobacters* in these products. Antimicrobial susceptibility testing shows increasing resistance to first‐line antibiotics used in clinical and veterinary settings. Detection of virulence and resistance genes showed that all targeted genes were found among *Arcobacter* strains. Then, handling raw milk and its direct consumption may expose humans to dangerous zoonotic agents such as *Arcobacter* species. These results also highlight the need for regular surveillance of *Arcobacter* strains in milk and milk products in Iran.

## ETHICS STATEMENT

This study was approved by the Shahrekord Branch, Islamic Azad University Ethical Committee.

## AUTHOR CONTRIBUTION

All authors read and approved the final manuscript.

## CONFLICT OF INTEREST

The authors declare no conflict of interest.

## Data Availability

All data generated or analysed during this study are included in this article. The datasets used and/or analysed during the current study are also available from the corresponding author.
